# Altered circadian genes expression in breast cancer tissue according to the clinical characteristics

**DOI:** 10.1371/journal.pone.0199622

**Published:** 2018-06-29

**Authors:** Monika Lesicka, Ewa Jabłońska, Edyta Wieczorek, Barbara Seroczyńska, Anna Siekierzycka, Jarosław Skokowski, Leszek Kalinowski, Wojciech Wąsowicz, Edyta Reszka

**Affiliations:** 1 Department of Molecular Genetics and Epigenetics, Nofer Institute of Occupational Medicine, Lodz, Poland; 2 Department of Medical Laboratory Diagnostics and Bank of Frozen Tissues and Genetic Specimens, Medical University of Gdansk, Gdansk, Poland; 3 Department of Surgical Oncology, Medical University of Gdansk, Gdansk, Poland; 4 Biobanking and Biomolecular Resources Research Infrastructure (BBMRI.PL), Gdansk, Poland; 5 Department of Biological and Environmental Monitoring, Nofer Institute of Occupational Medicine, Lodz, Poland; University of North Carolina at Chapel Hill School of Medicine, UNITED STATES

## Abstract

Breast cancer has a multifactorial etiology. One of the supposed and novel mechanisms is an alteration of circadian gene expression. Circadian genes play a crucial role in many physiological processes. These processes, such as genomic stability, DNA repair mechanism and apoptosis, are frequently disrupted in breast tumors. To assess the significance of circadian gene expression in breast cancer, we carried out an analysis of *CLOCK*, *BMAL1*, *NPAS2*, *PER1*, *PER2*, *PER3* and *CRY1*, *CRY2*, *TIMELESS*, *CSNK1E* expression by the use of the quantitative Real-Time PCR technique in tumor tissue and non-tumor adjacent normal tissue sampled from 107 women with a newly diagnosed disease. The obtained data were compared to the clinical and histopathological features. *PER1*, *PER2*, *PER3*, *CRY2* were found to be significantly down-expressed, while *CLOCK*, *TIMELESS* were over-expressed in the studied tumor samples compared to the non-tumor samples. Only gene expression of *CRY1* was significantly down-regulated with progression according to the TNM classification. We found significantly decreased expression of *CRY2*, *PER1*, *PER2* genes in the ER/PR negative breast tumors compared to the ER/PR positive tumors. Additionally, expression of *CRY2*, *NPAS2* genes had a decreased level in the poorly differentiated tumors in comparison with the well and moderately differentiated ones. Our results indicate that circadian gene expression is altered in breast cancer tissue, which confirms previous observations from various animal and in vitro studies.

## Introduction

Circadian rhythm is the most important cyclic changes system in all living organisms across the world, generated by the endogenous circadian clock. Circadian rhythm plays a crucial role in the maintenance of genomic stability and homeostasis. Globalization and fast-paced life of the contemporary world contribute to widespread changes in circadian coordination, resulting in civilizational diseases including metabolic syndromes, cardiovascular diseases, ageing, psychological disorders, neurodegenerative diseases as well as many types of cancer [1].

The core pacemaker of circadian rhythm is located in the suprachiasmatic nucleus of the hypothalamus, which synchronizes with both: endogenous and external factors [2]. Peripheral control systems are located in many tissues including breast tissue [3]. The molecular circadian clock at a transcriptional level consists of two groups of genes: positive and negative regulators. Transcriptional factors, i.e., *CLOCK*, *BMAL1*, and *NPAS2* that form heterodimers CLOCK/BMAL1 and NPAS2/BMAL1 belong to the first group. These heterodimers initiate transcription via E-boxes in promoters of the second group, i.e.: *CRY1*, *CRY2*, *PER1*, *PER2*, *PER3*. CRYs and PERs proteins inhibit heterodimers complexes as a negative loop of the circadian clock. The auxiliary loop consisting of REV-ERB/ROR ensures stabilization of this system [4, 5]. The described above physiological processes occur consecutively in each normal cell; however, in cancerous cells, this order is not preserved [6, 7]. Cancer disease with a complex multi-step etiology is manifested by abnormal physiology on a cellular level. Currently, there is more and more evidence, that circadian clock genes expression may play a crucial role in mammary gland pathophysiology. Circadian rhythm disruption may initiate various tumor-related processes [8]. A relevant process for cells, i.e. cell cycle is indirectly regulated by clock genes. Therefore, alterations of this process are manifested by abnormal cell division in many types of cancer [9]. This is probably caused by the disrupted synthesis of melatonin—nocturnal hormone, due to a permanent exposure to artificial light during the night, which particularly relates to night shift workers [10]. Clock genes belong to tumor suppressor genes that can control cell cycle, apoptosis, DNA damage and repair system [11]. The basic genetic regulation of the circadian clock is highly conserved across living organisms. The first evidence that deregulation of circadian rhythm due to environmental factors initiates breast carcinogenesis was hypothesized by Hamilton in 1969 [12]. Disruptions of the circadian gene expression pattern as an early event that leads to breast cancer initiation have been hypothesized by Stevens [13].

Disruption of circadian machinery in the human body may be influenced by three factors such as genetic defects, ageing of the body and lifestyle, also including shift work system [6, 14]. These factors together or individually may lead to dysregulation of the circadian clock, which is manifested by an altered cell cycle, reduced apoptosis, altered cell metabolism and disturbed melatonin synthesis. All the mentioned factors reveal a potential link with breast carcinogenesis [15]. An altered pattern of circadian gene expression is observed in the case of *in vitro* models, rodent animals as well as in human genetic association studies [16]. Clock genes alterations in mammary epithelium may lead to cell cycle dysregulation, which is caused by aberrant cell divisions, which can be associated with tumor progression and a more aggressive type of breast cancer tumors [17].

The main objective of the study was to present expression pattern of ten core clock genes (*CLOCK*, *NPAS2*, *BMAL*, *CRY1*, *CRY2*, *PER1*, *PER2*, *PER3*, *TIMELESS*, and *CSNK1E*) in breast cancer tissues in comparison to the paired non-tumor adjacent tissue according to the histological and clinical features, including. estrogen (ER), progesterone (PR) receptor status, HER2 receptor status, molecular type of the breast cancer, Tumor Node Metastasis stage (TNM) classification and grading.

## Materials and methods

### Ethics declaration

This study was conducted in compliance with the Declaration of Helsinki and was approved by relevant Local Ethics Committees (Independent Ethics Committee at Medical University of Gdansk, resolution No. NKEBN/781/2005, and Ethical Institutional Review Board at the Nofer Institute of Occupational Medicine resolution No. 20/2014). We have obtained the written consent of participants to deposit their tissue samples in the Bank of Frozen Tissues and Genetic Specimens of the Medical University of Gdansk and forward for further analyzes.

### Study population

We analyzed 107 Polish women, all Caucasian origin with a newly diagnosed breast cancer and without previous chemotherapy. The patients were admitted to the Clinic of Oncologic Surgery at the Medical University of Gdansk over the years 2006–2015 and divided into four groups according to the TNM classification. During the tumor removal surgery, tumor tissue and tumor-adjacent normal tissue (0.6 cm x 0.6 cm) were collected from each patient, then the pairs of tissue samples were frozen in -70°C and deposited in Bank of Frozen Tissues and Genetic Specimens of Medical University of Gdansk. Finally, they were shipped to the Nofer Institute of Occupational Medicine in Lodz (NIOM) for a further molecular analysis. The patient’s characteristics are presented in Table 1. All the tissue specimens were evaluated by pathologists to confirm the diagnosis of both tumor and adjacent normal tissue that was collected close to the resection margins. Subsequently, samples were specified according to estrogen receptor status (ER), progesterone receptor status (PR), Human Epidermal Growth Factor Receptor 2 (HER2) receptor status. Antigen retrieval and staining were performed by immunohistochemistry using the automatic devices: BenchMark GX (Ventana Medical Systems, Roche; Tucson, Arizona, AZ, USA) for all mentioned receptors and additionally fluorescent in situ hybridization analysis (only for equivocal HER2 positive samples with IHC 2+ score). Following criteria were included: estrogen receptor expression was determined as positive—IHC ranged from 4 to 8 as the intensity of the expression of ER receptor and as negative—IHC ranged from 0 to 3 as the intensity of the expression of ER receptor. Moreover, patients underwent a comprehensive histo-clinicopathological and post-surgical examination of tumor size (T-status), lymph node (N) status, and tumor grade (G1- well differentiated; G2- moderately differentiated; G3- poorly differentiated) histological type of breast cancer (*Carcinoma ductale*, *Carcinoma lobulare*). Additionally, molecular type of breast cancer were determined, according to this characteristics patients were divided into four molecular subtypes of breast cancer including Luminal A (estrogen-receptor and/or progesterone-receptor positive, HER2 negative) Luminal B (estrogen-receptor and/or progesterone-receptor positive HER2 positive) Basal-like (estrogen-receptor and progesterone-receptor negative, HER2 negative), HER2-enriched (estrogen-receptor and progesterone-receptor negative, HER2 positive).

**Table 1:** Characteristics of the study breast cancer patients.

Characteristics	All the patients n = 107 (100%)	*%*
**Age (mean±SD)**	60.22±13.17	
**BMI (mean±SD)**	26.84±4.57	
**Smoking**		
No smokers	80	74.77
Ever smokers	26	24,20
Unknown	1	0,93
**Estrogen receptor status (ER)**		
ER-positive	76	71.03
ER-negative	27	25.23
unknown	4	3.74
**Progesterone receptor status (PR)**		
PR-positive	68	63.55
PR-negative	35	32.71
unknown	4	3.74
**HER2-receptor status**		
HER2-positive	40	37.38
HER2-negative	59	55.14
unknown	8	7.48
**Molecular type**		
Luminal A	43	40.18
Luminal B	32	29.9
Basal-like	16	14.95
HER2-enriched	7	6.54
unknown	9	8.41
**Tumor grade**		
G1+G2	9+58	62.61
G3	37	34.57
unknown	3	2.82
**Menopausal status**		
Premenopausal	23	21.5
Postmenopausal	84	78.5
unknown	0	0
**TNM classification (Tumor, Nodes, Metastasis)**		
T1N0	30	28.04
T1N1-3	24	22.43
T3N0	23	21.49
T3N1-3	30	28.04
**Carcinoma Type**		
*Carcinoma ductale*	69	64.48
*Carcinoma lobulare*	17	15.88
Mix types of carcinomas	21	19.64

### RNA isolation procedures

Total RNA was isolated by the use of the RNeasy Lipid Tissue KIT (Qiagen, Hilden, Germany,) according to the manufacturer’s protocol. The amount of the total extracted RNA was determined using the spectrophotometer method—microplate (Multiskan^™^ GO Microplate Spectrophotometer, Thermo Fisher; Waltham, Massachusetts, MA, USA).

### Gene expression analysis

Expression analysis of *CLOCK*, *NPAS2*, *BMAL*, *CRY1*, *CRY2*, *PER1*, *PER2*, *PER3*, *TIMELESS* and *CSNK1E* in the breast tumor tissue and the adjacent tumor tissue was performed by means of quantitative Real-Time PCR (qRT-PCR). Primers were designed with BEACON DESIGNER 7.01 (PREMIER Biosoft International, Palo Alto, CA, USA). Expression of core circadian genes was quantified with the FastStart Essential DNA Green Master (Roche, Indianapolis, IN, USA) and using glyceraldehydes 3-phosphate dehydrogenase (*GAPDH*) and 60S acidic ribosomal protein P0 (*RPLP0*) as endogenous controls which present stable expression levels in RNA isolated from the breast tumor tissue and adjacent normal breast tissue samples. cDNA of the tumor and tumor-adjacent samples were synthesized on 200ng RNA with Transcriptor First Strand cDNA Synth, Kit 200 (Roche, Indianapolis, IN, USA). All the gene expression procedures fulfilled the MIQE guidelines (Minimum Information for Publication of Quantitative Real-Time PCR Experiment). PCR efficiency was calculated using dilutions of five pooled cDNA samples, which were reversely transcribed from the total human RNA isolated from the breast tissue samples. Each of the predesigned primer sets should meet the criterion of 90% qRT-PCR efficiency. Randomly selected samples were simultaneously amplified on the same plate to determine the inter-assay coefficients of variability (CV). The samples were assayed on the PTC-200 thermocycler (MJ Research, Bio-Rad Laboratories, Inc. Hercules, California, CA, USA) (cDNA synthesis) and LightCycler^®^ 96 (Roche, Indianapolis, IN, USA) (qRT-PCR assay). In order to calculate gene expression, we used reference gene-normalized relative quantification with efficiency correction for the target and reference genes (based on Livak method). Values of the Mean Normalized Expression (MNE) were log10 transformed and multiplied by 1000 to obtain a normal distribution of variables.

### Statistical analysis

Continuous variables were expressed as means with standard error or medians with interquartile ranges. Gene expression levels in the breast cancer tissue samples were compared with the paired adjacent non-tumorous tissue samples, calculated using the mean normalized expression and log-transformed so as to achieve normal distribution. Normality was assessed using the Shapiro–Wilk W-test. Comparison between the multiple groups was performed using the analysis of variance (ANOVA), followed by the in between-group comparisons with the Tukey HSD test if ANOVA yielded a significant difference. Differences among the paired tissue sets were evaluated by the paired t-test or the Wilcoxon signed rank sum test. Additionally, p-value has adjusted for age, BMI, smoking status, menopausal status by linear regression. Significance was declared for p levels less than 0.05. Statistica12.0 (StatSoft, Tulsa, OK, USA) was used for the statistical analysis.

## Results

### Subjects characteristics

Table 1 shows clinicopathological features of the patients with primary breast cancer without previous chemotherapy. A total of 107 patients (60.22±13.17 years of age and BMI 26.84 ±4.57) were enrolled into the study. Seventy-four and seventy-seven percent of the patients were non-smokers (n = 80) and 24.20% were smokers (n = 26). One patient had an unknown smoking status (0.93%). Majority of the breast cancer patients were classified as ER, PR positive (n = 76, 71.03%; n = 68, 63.55%, respectively) in comparison to the patients with negative ER, PR status (n = 27, 25.23%; n = 35, 32.71, respectively). This is consistent with the literature, which states that about 70% of breast cancer patients are ER, PR positive. Four women did not specify their hormonal receptor status. Fifty-nine patients were HER2 negative (55.14%), while the HER2 positivity rate was 37.38% (n = 40). TNM was specified for all the patients. We divided them into four groups: T1N0 (n = 30; 28.04%); T1N1-3(n = 24; 22.43%); T3N0 (n = 23; 21.49%); T3N1-3 (n = 30; 28.04%).

### Disrupted circadian gene expression in the breast cancer tissue samples compared to the non-tumor tissue samples

In order to determine whether circadian genes expression, including *CLOCK*, *NPAS2*, *BMAL*, *CRY1*, *CRY2*, *PER1*, *PER2*, *PER3*, *TIMELESS*, and *CSNK1E*, was altered in the breast tumor samples, we performed quantitative Real-Time PCR with intercalating SYBR Green in the 107 pairs of the tumor tissue and non-tumor adjacent normal breast tissue samples. We did not obtain detectable transcript levels for all the tissue samples in the total analysis, thus, the number of tissue specimens for the statistical analysis was limited and it is included in presented tables. (Tables 2 and 3). Our results indicated that the analyzed genes presented different expression patterns compared to the adjacent normal breast tissue samples. The strong statistical significance of the correlation between the investigated genes was observed in the case of the tumor tissue samples in comparison with the correlation analysis between the genes in the adjacent normal tissue samples and between types of the tissues. The transcript level was significantly up-regulated in the breast cancer tissue samples in comparison with the adjacent normal tissue samples for *CLOCK* (β-coefficient 0.219, p = 0.002) and *TIMELESS* (β-coefficient 0.298, p<0.0001). A significantly decreased mRNA level in the breast cancer tissue samples was observed for *CRY2* (β-coefficient -0.424, p<0.0001), *PER1* (β-coefficient -0.371, p<0.0001), *PER2* (β-coefficient -0.149, p = 0.037) and *PER3* (β-coefficient -0.231, p = 0.001). However, we did not observe a statistically significant correlation in the gene expression of *BMAL1*, *CRY2*, *NPAS2*, *CSNK1E* in the tissue pairs (Table 2).

**Table 2:** Comparison of circadian gene expression in the breast cancer tissue samples and the tumor-adjacent normal tissue samples.

Gene	Tumor-adjacent normal tissue samples Mean^a^±SEM	Breast cancer Tissue samples Mean^a^±SEM	N^b^	p-value^c^	β- coefficient	p-value^d^
***CLOCK***	1.84±0.035	2.03±0.04	88	0.002	0.219	0.002
***BMAL1***	1.78±0.037	1.81±0.037	78	0.94	0.062	0.410
***CRY1***	2.10±0.039	2.15±0.046	87	0.41	0.064	0.380
***CRY2***	2.36±0.042	1.94±0.049	94	<0.0001	-0.424	0.000
***PER1***	2.07±0.05	1.63±0.059	96	<0.0001	-0.371	0.000
***PER2***	2.12±0.039	2.00±0.045	94	0.01	-0.149	0.037
***PER3***	2.44±0.039	2.21±0.055	94	<0.0001	-0.231	0.001
***NPAS2***	1.32±0.072	1.47±0.078	68	0.08	0.109	0.164
***CSNK1E***	2.75±0.037	2.80±0.05	96	0.4	0.052	0.466
***TIMELESS***	1.31±0.058	1.66±0.061	86	<0.0001	0.298	0.000

^a^Mean of the Mean Normalized Expression (MNE).

^b^ The N reflects factual tissue pairs where we obtain data in Real-Time PCR analysis.

^c^Paired Student t-test or Wilcoxon test.

^d^*p*-value adjusted for age, BMI, smoking status, menopausal status by linear regression.

**Table 3:** Circadian gene expression in the breast cancer tissue samples according to the TNM classification.

Gene	T1N0	*N*^b^	T1N1-N3	*N*^b^	T3N0	*N*^b^	T3N1-N3	*N*^b^	*p-value*^c^
	Mean ±SEM^a^		Mean ±SEM^a^		Mean ±SEM^a^		Mean ±SEM^a^		
***CLOCK***	1.93±0.063	29	2.03±0.1	24	2.08±0.109	22	2.08±0.06	30	0.45
***BMAL1***	1.73±0.04	28	1.88±0.07	23	1.79±0.103	20	1.88±0.062	28	0.26
***CRY1***	2.05±0.074	29	2.04±0.093	24	2.17±0.103	21	2.32±0.071	27	<0.001
***CRY2***	2.06±0.082	29	1.87±0.096	24	1.86±0.136	22	1.93±0.073	30	0.43
***PER1***	1.79±0.102	29	1.65±0.122	24	1.42±0.141	22	1.59±0.095	30	0.16
***PER2***	1.94±0.059	29	1.95±0.092	23	1.99±0.119	22	2.1±0.082	30	0.52
***PER3***	2.05±0.084	29	2.23±0.136	23	2.25±0.136	22	2.32±0.080	30	0.24
***NPAS2***	1.51±0.11	25	1.53±0.154	22	1.1±0.144	18	1.62±0.129	24	0.06
***CSNK1E***	2.65±0.082	29	2.87±0.114	24	2.78±0.115	22	2.89±0.079	30	0.22
***TIMELESS***	1.5±0.08	29	1.81±0.118	24	1.61±0.162	22	1.76±0.095	30	0.16

^a^ One-way ANOVA test or Kruskal–Wallis test

^b^ The N reflects factual type of tissue sample where we obtain data in Real-Time PCR analysis

^c^ Mean of the Mean Normalized Expression (MNE)±SEM

### Circadian gene expression according to the TNM classification

To investigate whether circadian genes expression is associated with the tumor stage, we analyzed the breast cancer tumor tissue samples according to the TNM classification (Table 3). The analysis included four groups consisting of T1N0–29 samples, T1N1-3–24 samples, T3N0–22 samples and T3N-3–30 samples. A significant difference (p<0.001) was reported only for *CRY1*. Additionally, the analysis showed marginal statistical significance for *NPAS2*. In general, some changes in the transcript level between the groups were observed; however, they were not statistically significant.

### Decreased circadian gene expression in the negative ER and PR breast cancer tissue samples

**Fig 1:**
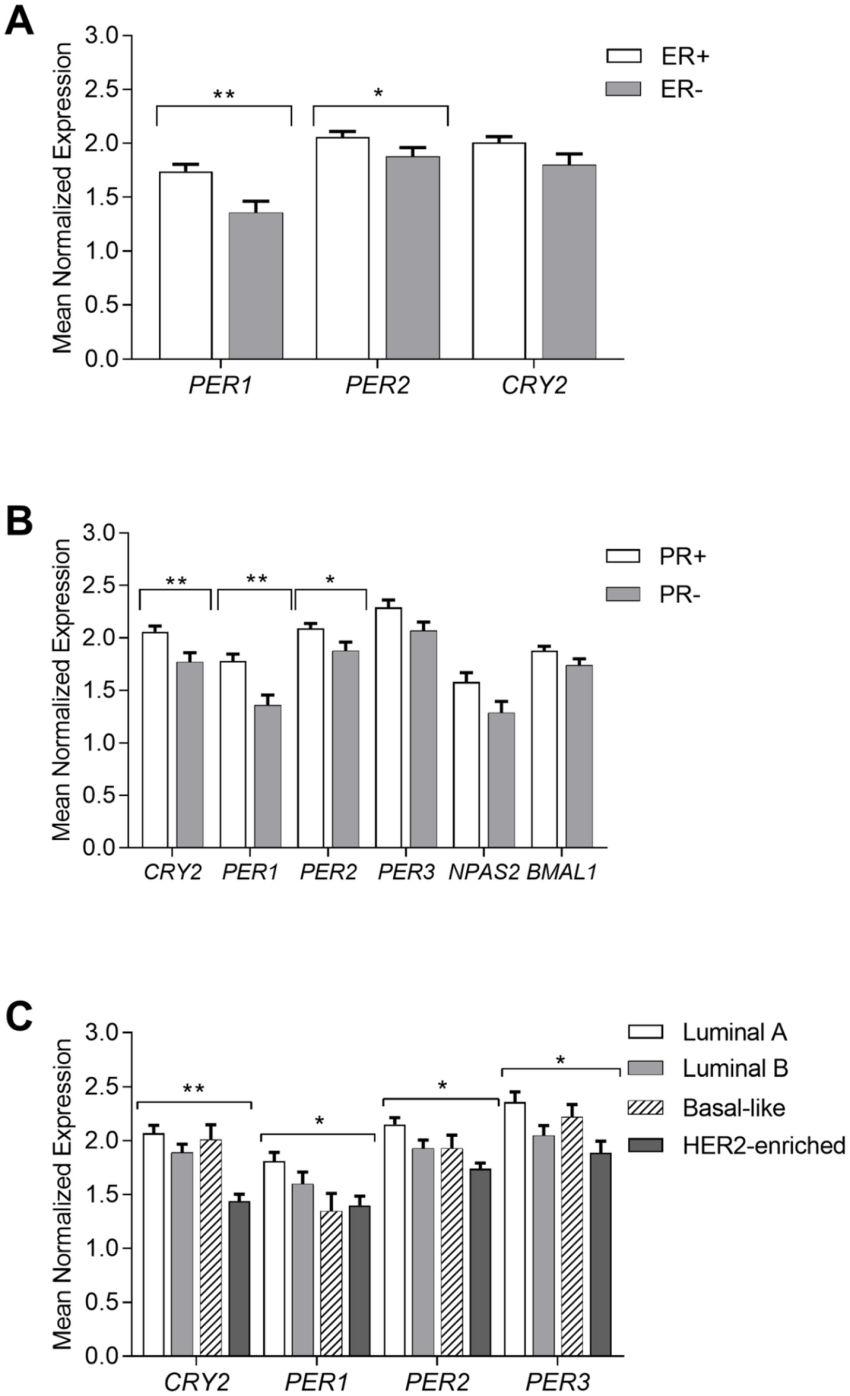
Circadian gene expression analysis according to the hormonal status and molecular type in the breast cancer tissue samples. **(**A) Estrogen receptor status. (B) Progesterone receptor status. (C) Molecular type of breast cancer. Results presented as a mean of the Mean Normalized Expression (MNE)±SEM; statistical significance marked as *—p <0.05; **—p <0.01 obtained by Student’s t-test or One way ANOVA test.

Hormone receptor status is an essential clinical factor in the evaluation of breast cancer diagnosis which determines a proper therapy. We carried out an analysis of each circadian gene expression according to the ER/PR status in almost each breast cancer tissue sample. Associations for few genes were found. The ER-negative (ER-) breast cancer tissue samples had significantly lower *PER1* gene expression than the ER-positive breast cancer tissue samples (ER+) (β-coefficient -0.291 p = 0.005). A similar marginal significant difference was observed for *CRY2* (β-coefficient -0.178 p = 0.088). For other genes, we did not observe any changes in the transcript levels, between ER- and ER+ (Fig 1). A further comparison by PR receptor status showed that the patients with PR-negative tumors (PR-) had significantly lower levels of *CRY2*, *PER*,*1 PER2 (*β-coefficient -0.266; p = 0.008; β-coefficient -0.335, p = 0.001; β-coefficient -0.217; p = 0.034; respectively) and marginally significant lower levels of *BMAL1*, *PER3*, *NPAS2* gene expression (β-coefficient -0.177 p = 0.09, β-coefficient -0.187; p = 0.068, β-coefficient -0.215 p = 0.057, respectively) than the patients with PR positive tumors (PR+) (Fig 1). This may suggest that tumor suppressing circadian genes (*PER1*,*2*,*3*, *NPAS2*) are particularly relevant for ER/PR negative tumorigenesis. All the specimens were classified into the molecular groups according to the presence of receptors on their surface: Luminal A, Luminal B, basal-like, HER2 overexpressed. mRNA level of each gene was compared to the receptor status including ER, PR, and HER2 (ER/PR positive, HER2 negative). The data analysis showed a significant difference between the molecular types for *CRY2*, *PER1*, *PER2*, *PER3* (p<0.01, p = 0.04, p = 0.03, p = 0.03, respectively) (Fig 1). For other genes, no significant results were obtained (data not shown).

### Decreased circadian gene expression in the positive HER-2 breast cancer tissue samples

**Fig 2:**
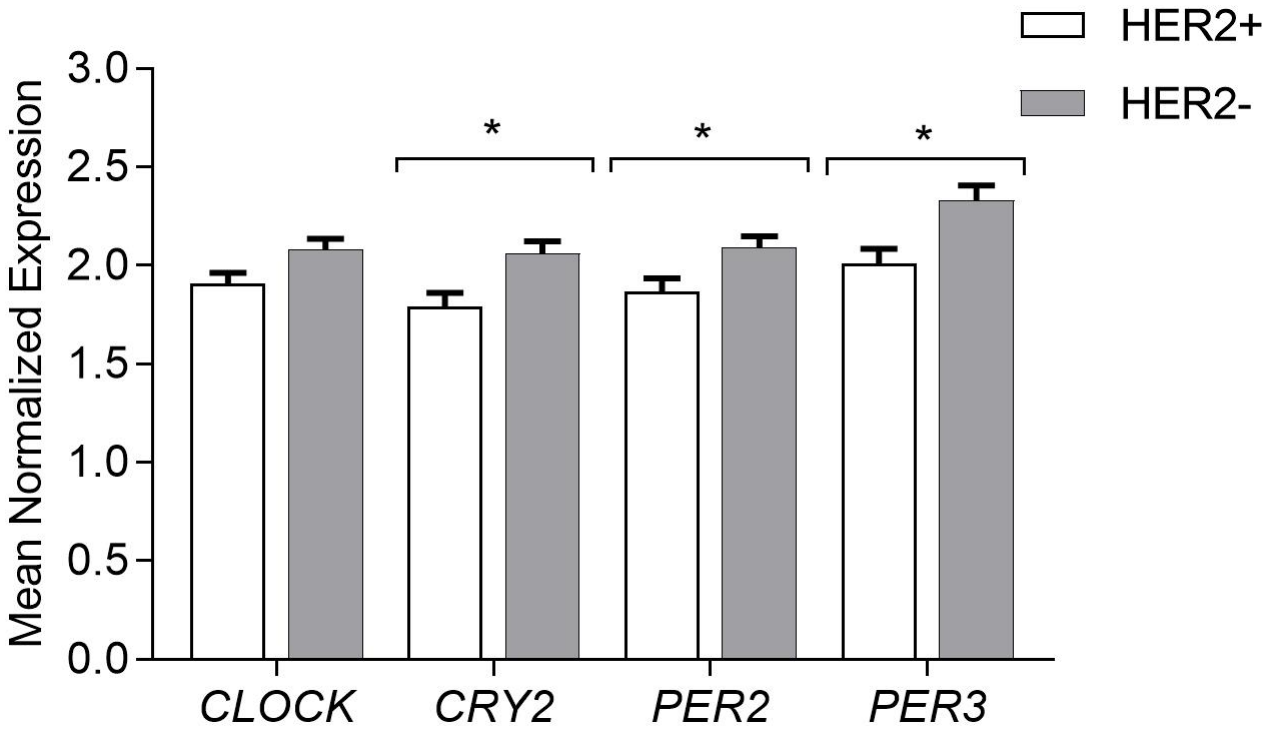
Circadian gene expression according to the HER-2 receptor status. Results presented as mean of the Mean Normalized Expression (MNE)±SEM statistical significance marked as *—p <0.05; **—p <0.01 obtained by Student’s t-test.

Expression status of proto-oncogene human epidermal growth factor receptor -2 (HER-2) in cancer cells determines the aggressiveness of breast cancer among the patients. It is used as a prognostic and predictive marker in breast cancer treatment. Our data was correlated with the HER-2 receptor status. Significant associations were observed for *CRY2*, *PER3*, *PER2 (*β-coefficient -0.266 p = 0.009, β-coefficient -0.289 p = 0.005, β-coefficient -0.217 p = 0.039, respectively). mRNA level of those genes was decreased in the HER2 positive tumors (Fig 2).

### Expression of circadian genes according to the histological type of carcinoma

**Fig 3:**
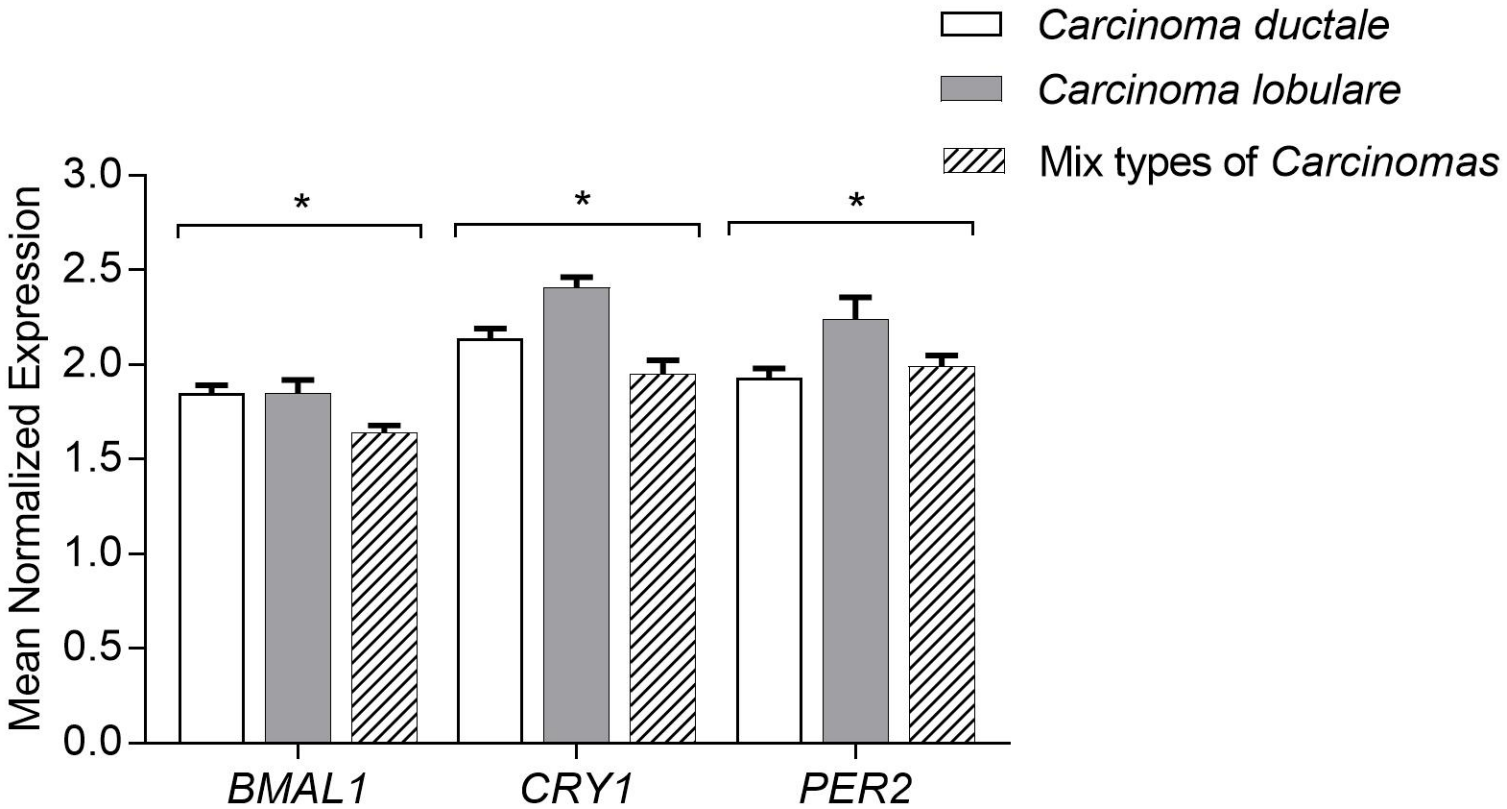
Circadian gene expression analysis according to the histopathological classification of the breast cancer tissue samples. Mix type of carcinomas include: *Adenocarcinoma mucinosum*, *Carcinoma adenoides cysticum*, *Carcinoma ductale partim lobulare*, *Carcinoma metaplasticum mamame ac diferentiatio planoepitheliele*, *Carcinoma mucinosum*, *Carcinoma tubulolobulare*, *Carcinosarcoma*. Results presented as a mean of the Mean Normalized Expression (MNE)±SEM statistical significance marked as *—p <0.05; **—p <0.01 obtained by One-way ANOVA test.

All the analyzed samples were characterized by histopathological features. We divided the tissues into 3 groups–*Carcinoma ductale*, *Carcinoma lobulare* and mix type of carcinomas including *Adenocarcinoma mucinosum*, *Carcinoma adenoides cysticum*, *Carcinoma ductale partim lobulare*, *Carcinoma metaplasticum mamame ac diferentiatio planoepitheliele*, *Carcinoma mucinosum*, *Carcinoma tubulolobulare*, *Carcinosarcoma*. Significantly decreased transcript levels of *CRY1*, *PER2* were observed in *Carcinoma ductale* and mix types of carcinomas (Fig 3).

### Expression of circadian genes according to the tumor grading

**Fig 4:**
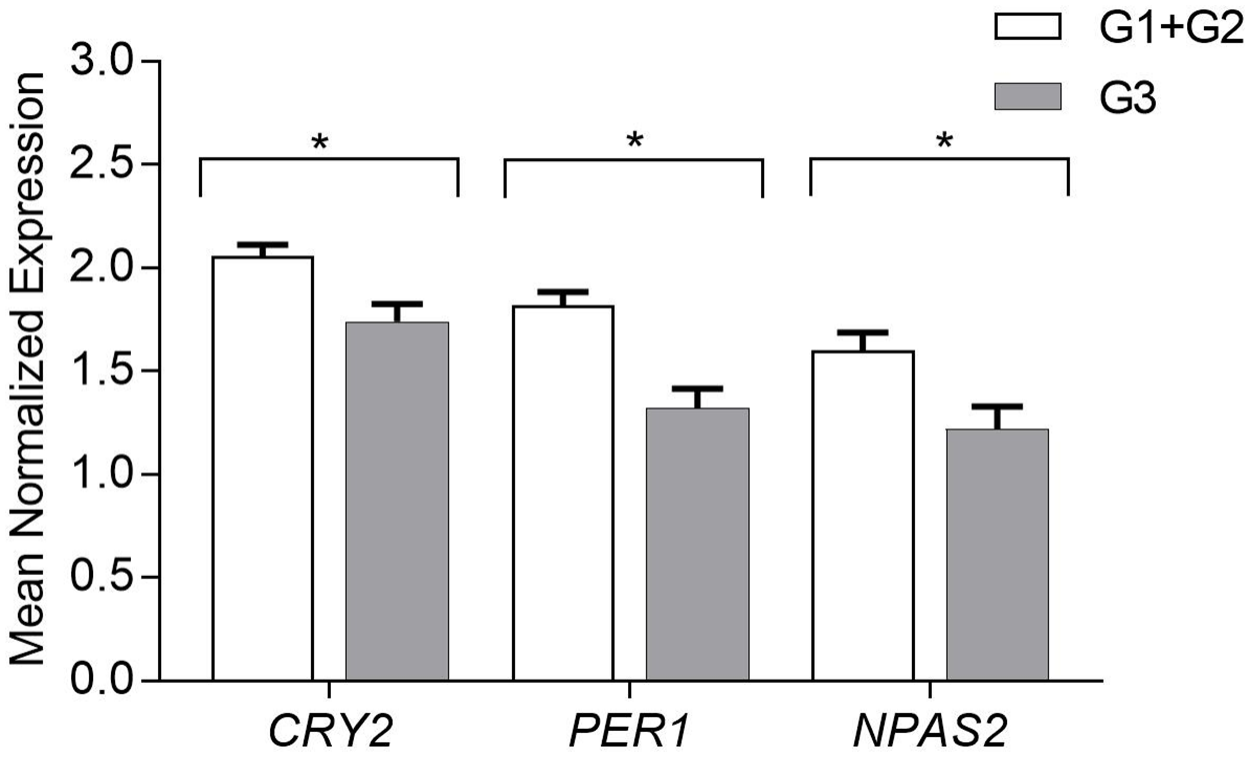
Circadian gene expression analysis according to the tumor grading of the breast cancer tissue samples. Results presented as a mean of the Mean Normalized Expression (MNE)±SEM; statistical significance marked as *—p <0.05; **—p <0.01 obtained by Student’s t-test.

According to the tumor grading, breast cancer tissue samples were divided into three groups: G1-Well differentiated, G2- moderately differentiated, G3-poorly differentiated. Due to the limited number of tissue samples in the G1 group, this group was combined with the G2 for the statistical analysis. Data analysis showed that genes *CRY2*, *PER1*, *NPAS2* had a decreased level of mRNA expression in the G3-poorly differentiated tumors in comparison with the G1- well and the G2-moderately differentiated tumors (β coefficient 0.28, *p =* 0.004; β coefficient 0.39 *p* = 0.00005; β coefficient -0,3 *p* = 0.009 respectively) (Fig 4).

## Discussion

In general, circadian genes are deregulated in breast cancer patients. Therefore, they may play an important role in cancer-related processes [16, 18–20]. So far studies suggest that the disruption of the circadian pattern may lead to vast consequences at a molecular level—previous evidence has shown that 2–10% of all mammalian genes display circadian oscillation [21] [22]. Our results support the hypothesis that disruption of clock genes in breast cancer may play an important role in the disease-related pathways. To elucidate the association between breast cancer and circadian rhythm, along with better understanding of breast tumorigenesis, we decided to correlate expression of the core clock genes with the clinical and histological characteristics. Circadian gene expression was detected in the paired tumor and non-tumor adjacent tissue samples obtained from the newly diagnosed breast cancer patients. Expression of some investigated circadian genes was significantly decreased in the cancerous tissue samples in comparison with the normal tissue samples from the breast cancer patients. The present data indicate that expression of *TIMELESS*, *CLOCK* in breast cancer tissue was over-expressed unlike the reduced transcript level of *CRY2* and *PER1*, *2*, *3*. So far, nine studies on circadian genes expression in human breast cancer tissues have been performed [18, 20, 23–29]. The first study on circadian gene expression in human breast cancer has been conducted by Chen *et*. *al*. Using the immunohistochemical assay they have found that PER proteins were deregulated in breast cancer tissue [18]. Similar observations have been pointed out by Kuo *et*. *al*. [20], who have found that homogeneous expression of PER2 was associated with lymph node metastasis and a poor prognosis [20]. Some studies have indicated similar results showing that genes *PER1*, *PER2*, *PER3*, and *CRY2* were down-expressed in breast cancer tissues. On the other hand, *CLOCK* and *TIMELESS* were found to be over-expressed in tumor tissue in comparison with normal adjacent breast cancer tissues [16].

Our results comply with the previous *in vitro* and animal studies related to circadian disturbances in breast cancer. Firstly, the described alterations in the functioning of circadian rhythm have been observed in animal and *in vitro* models. Downregulation of *Per1* and *Per2* in rodent models initiates higher cancer cell growth, but on the other hand, overexpressed *Per2* leads to apoptosis in mouse mammary carcinoma cell lines ETM6. *Per2* has a leading function in the circadian rhythm regulation [30]. Per2 directly controls c-myc—a gene responsible for proper functioning of the cell cycle [30]. *Per2* mutant mice have a deregulated *c-myc* and *p53* gene functions, leading to apoptosis reduction and cancer development after γ radiation exposure [30]. Furthermore, *PER* and *CRY* genes have been shown to be engaged in cell proliferation, including roles in DNA damage checkpoint control and regulation of important genes of cell cycle progression protein like *c-myc*, *Wee1*, *Myt1*, *Cyclin B*, *Cdc2* [31]. Due to the features specified above *PER* genes can act as tumor suppressor genes [30]. A few studies have shown a low level of *PER* and *CRY* expression in human breast cancer [18, 25, 26, 31–33]. Similar functions have been observed in human studies, where *PER1* and PER2 in normal cells promote apoptosis and act as tumor suppressor genes. In the case of breast cancer, a decreased transcript level of these genes is observed. *PER1*, *PER2* indirectly suppress c-myc transcription by inhibiting E-box mediated transactivation by BMAL1/NPAS2. The important role of circadian genes as one essential part of cell cycle machinery is stimulation of *ATM* and *CHK2* gene expression. Deficiency of *PER1* influences the altered expression of cell cycle checkpoint genes. It can be one of the early events of cancer initiation because of deregulations of these genes in breast cancer. *In vitro* studies on breast cancer cell lines show that the induced overexpression of *PERs* gene elicits cell cycle arrest, growth inhibition of cancerous cells and induces apoptosis. *CRYs* play a similar role in tumorigenesis activation via the cell cycle. Deficiency of *CRY* leads to the deregulated expression of *Wee-1*, *Cyclin D1* and reveals disrupted cell cycle regulation. Our experiment showed that expression of *CRYs*, *PERs* are statistically decreased in the breast cancer tissue samples compared to the normal tissues samples.

What is more, we compared expression levels of clock genes to the clinical and pathological features such as status of estrogen, progesterone and HER2 receptors, type of histological type of breast cancer, stage of tumor progression and tumor grading. A significantly increased *CRY1* expression level was observed in the most advanced stage—T3N1-3 in comparison with T1N0. This fact might be related to the defence mechanisms. According to the molecular type of breast cancer, the statistically significant lowest expression of *PER1* was observed in the triple negative breast cancer patients, while in the case of HER2-enriched, the lowest expression was observed for the negative loop of the central clock genes such as *CRY2*, *PER2*, *PER3*. We found a strong statistical association between the core circadian genes and hormone receptor status as well as tumor grading. They displayed significantly lower expression in the ER-negative, PR-negative and poor-differentiated tumors. These results are in agreement with the previously published studies showing how the loss of circadian gene expression may lead to the loss of cell proliferation control. Breast cancer patients with a negative hormone receptor status for ER and PR are less responsive to the treatment and have a worse course of the disease and progression. Thus, a suggestion that clock genes expression (like CRYs and PERs) and their higher activity in ER/PR positive tumors in comparison with ER/PR negative may imply an important relationship between ER, PR—signalling and circadian rhythm. This hypothesis is supported by recent studies conducted by Gery *et al*., who have found a link between ER status and *PER2* expression [33]. Cancer cell line MCF-7 with an ER-positive status treated with E2 had higher expression of *PER2* than cell lines without ER [33]. In the *PER2* promoter, they have found potential Estrogen Responsive Element (ERE) binding site at 356 bp. The same authors have found a significant relationship between the low level of *PER1* expression and the negative ER status [33]. Similar observations for *CRY* genes have been made by Hoffman *et al*. explaining why circadian genes expression is decreased in ER-negative tumors.

In addition, it is generally recognized that estrogen stimulation is one of the major initiators of breast tumorigenesis. When the ER status is taken into account, patients with ER-positive breast cancer possess a lower level of *CLOCK* expression than those with ER-negative tumors. This data imply that abnormal overexpression of *CLOCK* might be one of the early events in breast tumorigenesis [23]. Further experiments conducted by Xiao *et al*. have revealed opposite results. As much as 74% of examined ER-positive breast cancer tissues had a higher *CLOCK* expression than ER-negative samples measured by immunohistochemical assay [34]. A very interesting observation has been recently made by the same group. They have found that *CLOCK* is required for the proliferation of breast cancer. Those authors suggest that *CLOCK*, together with ER, could promote cancer cell proliferation. Breast cancer MCF-7 cell line with overexpression of *CLOCK* and ER showed higher growth than cell lines transfected with empty vector. This discovery indicates that it seems reasonable to assume that induction of *CLOCK* expression via ER is a core event in the proliferation of ER-positive breast cancer cells [34]. In our experiment, we did not observe similar directions. The transcript level did not differ according to the various tumor features. We only observed higher *CLOCK* expression in the tumor tissue samples in comparison with the non-tumor adjacent tissue samples.

There are not many studies on *TIMELESS* expression and its association with breast cancer. Our results are similar to those obtained by *Mao et al*. that indicate *TIMELESS* overexpression is different in breast tumor specimens compared to normal tissues. They have also identified a cancer-relevant network of transcripts with altered expression following *TIMELESS* knockdown, which contained many genes with known functions in cancer development and progression. Additionally, cell proliferation of *TIMELESS* knockdown MCF7 cell line was significantly decreased [35]. A recent study confirmed that *TIMELESS* levels in both breast cancer cell lines and tissues were significantly over-expressed. Upregulation of *TIMELESS* dramatically enhanced, while knockdown of *TIMELESS* suppressed the self-renewal of cancer stem cells (CSCs), cell invasion and migration abilities of breast cancer cells *in vitro* [36].

A general observation from this study is a statistically significant diminished expression level of *CRY2*, and *PERs* and an increased expression level of *CLOCK* and *TIMELESS* in the breast cancer tissue samples in comparison with the adjacent normal tissue samples. Another observation from our research indicates downregulation of CRYs and PERs in the ER/PR negative breast cancer tissue samples and in *Carcinoma ductale* histological type.

Circadian genes are involved in many physiological functions and their alterations could be manifested in various neoplasms, including breast cancer. According to the existing data regarding *CLOCK*-related modulation of breast cancer development, we shall focus on transcriptional and epigenetic regulation of the core clock genes assuming that: 1) circadian genes can modulate cancer suppression via caretaker, gatekeeper and landscaper genes and direct modification of chromatin structure; 2) expression of the clock genes seems to be epigenetically modulated, 3) there is cross-talk between the clock genes and ER status.
